# A case of total ophthalmoplegia associated with a COVID-19 infection: case report

**DOI:** 10.1093/omcr/omac050

**Published:** 2022-05-23

**Authors:** Doaa Hajjar, Dana Sultan, Abdullah Khalaf, Hussein Hesso, Ammar Kayyali

**Affiliations:** Department of Ophthalmology, Aleppo University Hospital, Aleppo, Syria

## Abstract

We are presenting a rare case of an acute complete external ophthalmoplegia with positive polymerase chain reaction (PCR) for SARS-CoV-2. Our case is the first case that depicts development of Tolosa-Hunt Syndrome (THS) following infection with COVID-19, with a challenging diagnosis and spontaneous improvement. A 65-year-old diabetic female presented with a complete external ophthalmopegia in the left eye and a severe left-sided headache. The PCR result for SARS-CoV-2 was positive. Brain and orbital computed tomography scan and magnetic resonance imaging were both unremarkable. We diagnosed the case as THS after ruling out other differential diagnoses. The patient refused to receive prednisone, so we had to observe her closely for 6 months during which period we recorded a spontaneous recovery. Acute ophthalmoplegia is a very challenging presentation. It needs full workup to exclude the wide range of differential diagnoses.

## INTRODUCTION

Since the start of the SARS-COV-2 pandemic, a number of both benign and vision-threatening neuro-ophthalmic manifestations have been documented in COVID-19 cases [1] of which we cite optic neuritis, nystagmus, visual field abnormalities, headache, diplopia, visual impairment, ocular pain, Miller Fisher syndrome and painful cranial neuropathies [2–4].

Cranial neuropathies, especially painful ophthalmoplegia, have a wide range of differential diagnoses, which makes the diagnosis challenging. Here, we have a rare case of complete ophthalmoplegia in a COVID-19-positive patient with unremarkable radiological findings and spontaneous remission through long-term observation.

The work has been reported in line with the SCARE criteria [5].

## CASE REPORT

A 65-year-old Caucasian female with uncontrolled diabetes mellitus was admitted to our ophthalmology department. She presented with left periocular pain, droopy eyelid and left ocular movement limitation for a period of 4 days. Two weeks before admission, she had had a severe left-sided headache unresponsive to sedatives, along with fatigue, dry cough and fever. The patient denied any history of trauma or recreational drug use.

**Figure 1:**
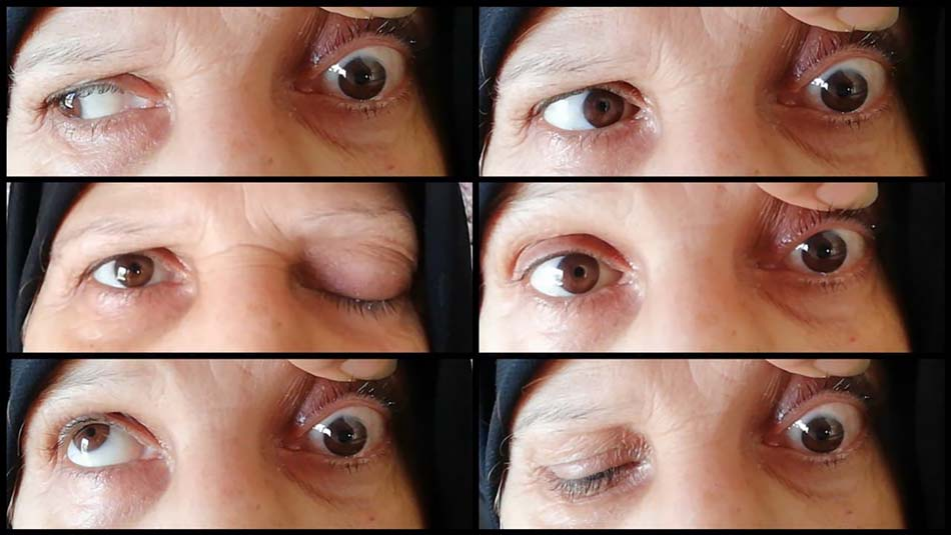
Photograph of the patient in six positions at the presentation, showing prominent ophthalmoplegia in the left eye.

Clinical examination revealed a complete external ophthalmoplegia on the left eye and total left ptosis with a mild chemosis. The pupil was semi-dilated and non-reactive to light. We also recorded mild proptosis on Hertel exophthalmometer (3-mm discrepancy between the two eyes). Uncorrected visual acuity was 0.6 (0.18 LogMAR), intraocular pressure was 16 mmHg and Ishihara color test was normal (Fig. 1).

The patient was conscious, oriented and responsive to stimuli. Tendon reflexes and muscle tone were all within normal limits. There were no meningeal or cerebellar signs. Ear, nose and throat examinations were within normal as well. Sensory system examination was totally normal at presentation. However, a left corneal hypoesthesia appeared 2 days after admission, which was consistent with an injury in the first branch of the fifth cranial nerve.

Laboratory studies (including CBC, PT, INR, D-Dimer, ferritin, urea, LDH, creatinine, SGOT, SGPT, sodium, potassium and calcium) were all within normal limits. By contrast, C-reactive protein was elevated (11.4 mg/l) and so was blood glucose (300 mg/dl).

Polymerase chain reaction for COVID-19 was positive, and brain and orbital computed tomography (CT) scan and magnetic resonance imaging (MRI) were both unremarkable. There was no sign of inflammation nor of a mass.

The diagnosis of Tolosa-Hunt Syndrome (THS) was made after the exclusion of other pathologies. The patient was admitted to the COVID-19 unit for close observation and blood glucose control and for the overall management of her COVID-19 symptoms. Brain MRI was normal upon its repetition after a week.

The patient’s status later improved, and she was discharged home with monthly reviewing.

On follow-up, her ocular symptoms and signs started to improve spontaneously 3 months after admission. A regression in the ptosis, a partial recovery in the third and fourth cranial nerves were detected. She could adduct, elevate and depress the left eye into primary and adduction positions.

**Figure 2:**
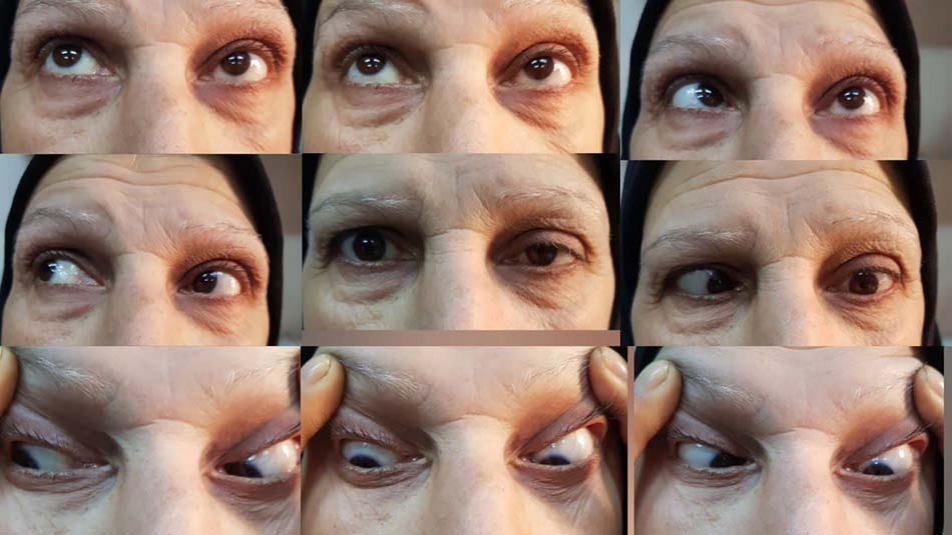
Photograph of the patient in nine positions after 5-month follow-up, showing improvement in the eye movements except for the sixth nerve in the left eye.

Corneal sensation was recovered 5 months after presentation. She could fully open her left eye. The pupil became round, regular and reactive to light, but she was unable to abduct the eye. This meant a complete spontaneous restoration of the third, fourth and first branches of the fifth cranial nerve (Fig. 2).

By the end of the 6-month period, all the involved nerve functions were restored except for the sixth nerve and the patient ended up with exotropia. At present, the patient is complaining of diplopia and is being prepared for strabismus surgery.

## DISCUSSION

Reports of neuro-ophthalmic complications in COVID-19 patients, such as cranial nerve paresis, optic neuritis and gaze palsy, are being increasingly described in the literature. This is a case of an infrequent presentation of painful complete ophthalmoplegia in a COVID-19-positive patient with radiologically normal imaging. We documented the natural course of the spontaneous remission of the neuropathies with no treatment.

Cranial nerve palsies with COVID-19 were reported. However, to our knowledge, this is the first case to report a concomitant four cranial nerves palsies, CN III, IV, V and VI.

Ophthalmoparesis has a wide range of differential diagnoses; multiple sclerosis, neoplasms, malignancies, giant cell arteritis, Guillain-Barre syndrome, THS, diabetes mellitus and cavernous sinus thrombosis.

In this specific case, there was acute severe complete external ophthalmoplegia, left corneal hypoesthesia, total ptosis and mild proptosis, with a history of severe headache and cough. We made the diagnosis of Tolosa-Hunt Syndrome (THS) after ruling out all the other pathologies.

Our patient had normal CT and MRI with no signs of masses or hyperintensity at presentation. They were also normal on repetition after a week. We could not use gadolinium for it was unavailable in our hospital.

Although the patient had uncontrolled diabetes, the clinical presentation was far from the classic presentation of diabetic ophthalmoplegia; it indicated a pathology in the orbital apex rather than only a diabetic ischemia. Our patient had severe and acute paralysis of three cranial nerves simultaneously, and all the nerves enter the cranial cavity through the orbital apex. As well as, the sensitive part of the fifth nerve was injured. Based on clinical presentation and MRI findings, we were able to rule out the most notable differential diagnoses and make the diagnosis of THS. The THS diagnosis is a triad of one or more episodes of unilateral orbital pain, paresis of one or more of the cranial nerves and granulomas in MRI or biopsy; which is sensitive for the diagnosis of the syndrome. However, the fact that over half of the reported cases had normal MRI renders the diagnosis dependent on the exclusion of other etiologies [6].

The patient was not given the corticosteroids treatment at her demand. She showed spontaneous remission and had to be monitored over a 6-month period.

Since our patient was positive for COVID-19, and there was no detection of any other possible etiology such as other infections, trauma, tumor or malignancy, we theorize that the SARS-COV-2 virus may have triggered an immunological reaction leading to the development of the ophthalmoplegia, which we believe is THS.

Our case is the first to report the development of complete acute ophthalmoplegia following COVID-19 infection with normal MRI and spontaneous improvement.

## References

[1] Grossman SN , Calix R, Tow S, Odel J, Sun L, Balcer L et al. Neuro-ophthalmology in the era of COVID-19: future implications of a public health crisis. Ophthalmology 2020;127:e72–4. 10.1016/j.ophtha.2020.05.004. 32387481 PMC7204645

[2] Gold DM , Galetta SL. Neuro-ophthalmologic complications of coronavirus disease 2019 (COVID-19). Neurosci Lett 2021;742:135531. 10.1016/j.neulet.2020.135531. 33248158 PMC7687583

[3] Luís ME , Hipólito-Fernandes D, Mota C, Maleita D, Xavier C, Maio T et al. A review of neuro-ophthalmological manifestations of human coronavirus infection. Eye Brain 2020;12:129–37. 10.2147/EB.S268828. 33154692 PMC7608548

[4] Costello F , Dalakas MC. Cranial neuropathies and COVID-19. Neurology 2020;95:195–6. 10.1212/WNL.0000000000009921. 32487714

[5] Agha RA , Franchi T, Sohrabi C, Mathew G, Kerwan A, Thoman A et al. The SCARE 2020 guideline: updating consensus surgical CAse REport (SCARE) guidelines. Int J Surg 2020;84:226–30. 10.1016/j.ijsu.2020.10.034. 33181358

[6] Mantia LL , Curone M, Rapoport A, Bussone G. Tolosa–Hunt syndrome: critical literature review based on IHS 2004 criteria. Cephalalgia 2006; 26:772–81. doi:10.1111/j.1468-2982.2006.01115.x. 16776691

